# Effect of personality traits on the oral health-related quality of life in patients with oral lichen planus undergoing treatment

**DOI:** 10.1007/s00784-020-03561-5

**Published:** 2020-09-14

**Authors:** Dvorak Gabriella, Rappersberger Klemens, Rausch-Fan Xiao-hui, Bruckmann Corinna, Hofmann Eva

**Affiliations:** 1 Clinical Division of Conservative Dentistry and Periodontology, University Clinic of Dentistry, Medical University of Vienna, Sensengasse 2a, 1090 Vienna, Austria; 2 Department of Dermatology and Venereology, Medical Institution Rudolfstiftung, Juchgasse 25, 1030 Vienna, Austria; 3 Competence Center for Empirical Research Methods, WU Vienna University of Economics and Business, Vienna, Austria

**Keywords:** OHIP, NE-OFFI, OLP, Mucosal disease, Therapeutic outcome, Therapy

## Abstract

**Objectives:**

The aim of this study was to evaluate the relationship between personality traits and perceived treatment success in oral lichen planus.

**Material and methods:**

A total of 53 patients with diagnosed oral lichen planus were evaluated at the time of diagnosis and along the course of their treatment. The visual analogue scale (VAS) was used for evaluating pain and burning sensation, along with an evaluation of the oral health-related quality of life (OHIP) and the clinical severity. In order to determine the personality trait, the NEO-FFI questionnaire was applied. Data were assessed with the statistical software Stata by a multiple linear regression.

**Results:**

A significant relationship between the two personality traits: “conscientiousness” and “extraversion” and a perceived improvement in oral lichen planus could be observed. The higher the “conscientiousness,” the better the perceived oral health-related quality of life. Furthermore, “extraversion” had a significant influence on the improvement in clinical severity index (*P* < 0.05).

**Conclusions:**

Personality traits, especially conscientiousness and extraversion, have a significant impact on the perception of therapeutic intervention in oral lichen planus.

**Clinical relevance:**

As personalized patient management is gaining importance and psychosocial factors play a significant role in mucosal diseases, the patient’s psychological profile should be considered in the oral lichen planus management.

## Introduction

Oral lichen planus (OLP) is a chronic inflammatory mucocutaneous disease. Prevalent in around 2% of the general population, it mainly affects women between 30 and 60 years of age. This immune disease is clinically characterized by different types of lesions, like reticular, so-called Wickham Striae, atrophic, or erosive areas, which may alternate [1]. Patients with symptomatic OLP require intensive care, as the disease may limit the perceived quality of daily life [2]. Specifically, pain stemming from erosive lesions may considerably impair food intake and oral hygiene accompanied by a high incidence of psychological problems. As of today, no curative treatment exists. Therapy focuses on the mitigation of symptoms, through drugs that counter inflammation and the underlying immune disorder. This symptomatic treatment may have side effects, like fungal infections or local irritations equally affecting oral health-related quality of life [3–5]. The uncertainty caused by OLP, as a potentially malignant disorder where the absence of available treatments to prevent such transformation [6], may stress the patients and affect their well-being [7].

OLP is assumed to be an autoimmune disease, yet the exact etiology is unknown. Psychological factors could play an important role as emotional stress is likely to be an associated risk factor. Furthermore, the burdens of chronic disease and persisting pain patients suffering from OLP are faced with require copying strategies. Personality traits influence behavior, compliance, and the perceived health-related quality of life, in mucosal diseases as elsewhere [8, 9]. Whether therapeutic success in the treatment of mucosal disease is associated with personality traits remains unproven so far. In prosthetic and orthodontic rehabilitation, the psychological profile should be considered in formulating the treatment plan since the patient’s satisfaction and acceptance are affected by personality traits [10–13].

The consequences of this condition on daily life, coping with the disease and quality of life, are all reflected by the Oral Health Impact Profile (OHIP). Also, the responsiveness of OHIP scores under therapy has been evaluated [14–16].

Clinicians should consider that psychological traits might play a relevant role in establishing individual limits of quality of life for specific treatments. Some measurable personality traits seem to have a considerable impact on individual satisfaction with therapy [17].

Since great importance is currently given to subjective outcome parameters, this study ought to provide additional data augmenting the possible contribution to clinical intervention.

We hypothesize that some personality traits are associated with higher scores of oral health-related quality of life in patients with OLP undergoing treatment.

## Material and methods

This was an observational cohort study carried out at the University Clinic of Dentistry of Vienna, Austria. Ethical approval was obtained from the Ethics Committee of the Medical University of Vienna. All patients gave written consent for their participation. Patients were eligible, if their diagnosis of oral lichen planus (OLP) was confirmed by histopathology and direct immunofluorescence assay; any removable dentures were correctly fitting; minimum age was 18 years; no nicotine abuse was reported. Patients were excluded with a malignant transformation, severe dysplasia in histopathology, carcinoma in situ, severe vitamin deficiency, pregnancy, age below 18 years, lactation period, nicotine abuse, the presence of asymptomatic OLP, or oral mucositis of other origins (e.g., drug intake).

### Patient recruitment

Patients with OLP were recruited at the outpatient clinic for mucosal diseases of the Division of Oral Surgery between October 2015 and August 2016. The diagnosis of OLP was made according to the clinical appearance and further confirmed by histopathology and direct immunofluorescence (IFT) analysis. All the tissue samples were taken by one specially trained surgeon (GD) and a specialist (KR) in immune dermatology and mucosal histopathology performed these analyses. The type of treatment (hyaluronic acid (Gengigel®), topical triamcinolon (Volon® A), tacrolimus, cyclosporin, photodynamic therapy (PDT), systemic glucocorticoids or deproteinized hemodialysate of dairy calves (Solcoseryl® ointment), the time between appointments, the clinical severity of OLP, and the subjective outcome parameters of oral health-related quality of life and pain intensity were assessed as well as the personality traits by questionnaires. The type of treatment was chosen individually depending on the patient’s symptoms in accordance with the treating dermatologist. In cases where the first-line treatment was not effective and patients came for a second opinion, calcineurin inhibitors were prescribed by a dermatologist (KR). Some of the patients suffering from lichen ruber planus of the skin or in other mucosal locations received systemic glucocorticoids. In mild cases or when patients refused immunosuppressant therapy, hyaluronic acid or hemodialysate was prescribed. All treatment options were discussed with the patient and the treating dermatologist, if available. Patients were recalled according to the clinical presentation every 3–6 months (90–200 days). In cases of subjective worsening, patients had the possibility to seek for an earlier appointment.

### Outcome parameters

#### Clinical severity index (Table 1) [18]

The clinical presentation and the therapeutic improvement of clinical signs may be assessed by a clinical criteria score. The Thongprasom score is such a clinical severity index evaluating disease intensity in many OLP studies [18]. The CSI was assessed at each appointment.

**Table 1:** Baseline characteristics of patients

Number	Age	Gender	Days between first and last visit	OHIP	VAS	CSI
1 2 3 4 5 6 7 8 9 10 11 12 13 14 15 16 17 18 19 20 21 22 23 24 25 26 27 28 29 30 31 32 33 34 35 36 37 38 39 40 41 42 43 44 45 46 47 48 49 50 51 52 53	58 79 71 69 70 58 79 31 68 77 54 60 45 55 33 57 47 61 60 59 47 64 68 47 74 58 49 59 49 54 74 49 64 43 56 24 37 50 21 54 36 72 69 40 42 61 74 67 71 56 60 58 53	F F F M F F F M F M F F F F M F F F F F F F F F M F F F F F F F F F F F F F F F F F F F F F F F F M F F M	1169 70 646 22 850 1713 42 511 581 924 28 14 840 532 560 525 28 280 504 842 21 91 357 203 525 1011 693 945 540 36 343 189 343 476 189 84 252 140 217 210 70 98 106 367 458 289 140 401 402 168 128 42 152	6 11 12 13 13 14 14 18 21 22 25 28 28 29 29 34 36 36 38 39 43 45 46 46 50 51 51 52 57 59 59 61 61 61 62 65 67 69 70 71 72 74 75 75 81 82 82 82 88 90 105 126 151	0 1.1 10 1 0 2.5 0 2.5 0 7.5 5 3 4.5 7.5 2.9 6.7 7.3 0 0 0 0 5.2 4.5 5.7 7.6 0 2.5 2.8 3.2 6.7 5 0 1.7 0 3.7 8 0 1.4 5.5 3 2.4 NA 5.2 6.7 3.1 9.2 7 8 0 2.5 1.5 6.9 3.4	3 4 3 3 3 4 1 1 1 3 3 4 2 4 2 4 4 NA 1 4 NA 2 3 2 4 2 1 1 1 5 2 2 1 1 1 1 2 4 NA 1 1 1 4 2 2 4 4 4 NA 3 2 4 3

0, no lesion/normal mucosa; 1, mild striae/no erythema; 2, white striae with atrophic area < 1 cm^2^; 3, white striae with atrophic area > 1 cm^2^; 4, white striae with erosion < 1 cm^2^; 5, white striae with erosion > 1 cm^2^.

#### The visual analogue scale (Table 1)

Subjective pain intensity evaluation is widely performed by a visual analogue scale (VAS) [19]. A customized absolute numeric scale ruler where patients rated their pain between 0 and 100 mm with a slider was used at each appointment. Scores of 0 imply “no pain,” while 100 means the “worst pain imaginable.” A recommendation for interpretation of the ratings suggest 0 to 4 mm to be read as no pain, 5 to 44 mm as mild pain, 45 to 74 mm as moderate pain, and 75 to 100 mm as severe pain. With this subjective evaluation method, it is possible to compare pain levels at every visit and to note score changes over time [20].

#### Oral Health Impact Profile (Table 2)

The OHIP is a cross-culturally adapted oral health-specific outcome measure recognized as an instrument suitable for the assessment of the oral health-related quality of life in cross-sectional as well as longitudinal studies. The Oral Health Impact Profile questionnaire German version (OHIP-G) is an oral health-related quality of life instrument containing 53 questions concerning the previous 4 weeks of the patients’ life. Answers to the 53 items of the OHIP-G are given on a Likert-type five-point scale (0, never; 1, hardly ever; 2, occasionally; 3, fairly often; and 4, very often). In this analysis, the total summary OHIP-G score was utilized, calculated by summing the score of the answers to each question. Higher scores indicated the lower oral health-related quality of life. Summing the responses to subsets of items created subscale scores. OHIP-49 is divided into seven different parts. The possible score range for each of these is as follows: “functional limitation” (9 items)—from 0 to 36; “physical pain” (9 items)—from 0 to 36; “psychological discomfort” (5 items)—from 0 to 20; “physical disability” (9 items)—from 0 to 36; “psychological disability” (6 items)—from 0 to 24; “social disability” (5 items)—from 0 to 20; “handicap” (6 items)—from 0 to 24; and finally “overall OHIP score” (49 items)—from 0 to 196. The German version has additional questions “DT” (4 items)—from 0 to 16. Four dimensions (psychosocial impact, orofacial pain, oral functions, and appearance) served as principal components of oral health-related quality of life. The dimensions were calculated according to 21 stable rotational components as described previously.

**Table 2:** OHIP mean of the differences in the summary score, subscale score, and subdimension score changes between the first and last visit

Scale	Mean	Standard deviation
OHIP-G	− 0.2	0.5
Functional limitation	− 0.1	0.5
Social disability	− 0.1	0.5
Handicap	− 0.0	0.5
Psychological disability	− 0.3	0.6
Physical disability	− 0.2	0.5
Psychological discomfort	− 0.6	0.9
Physical pain	− 0.4	0.9
DT	− 0.1	0.6
Oral function	− 0.0	0.8
Psychosocial impact	− 0.2	0.6
Orofacial pain	− 0.5	1
Appearance	− 0.6	0.9

The OHIP-G was assessed at each appointment.

#### Neuroticism Extraversion Openness Five Factors Inventory

This is an instrument to determine personality traits containing 60 questions. It analyzes five main behavior domains (neuroticism, extraversion, openness, agreeableness, and conscientiousness). Each of these domains is examined through 12 questions to be answered by one of the following Likert-type answers: “strong disagreement,” 0; “disagreement,” 1; “neutral,” 2; “agreement,” 3; or “strong agreement,” 4.

Data processing and scoring procedures were performed using appropriate software provided with the instrument forms. Scores were calculated by specific algorithms based on the population characteristics (country, region). The average ratings for each domain in NEO-FFI-R were related to OHIP scores to verify the influence of psychological profiles.

### Statistical analysis

For a medium effect size of *f*^*2*^ = 0.15, statistical power of 0.85, 17 predictor variables (sex, age, denture, missing teeth, CSI, 7 types of treatment, neuroticism, extraversion, openness, agreeableness, conscientiousness), and a probability level *P* = 0.05 at least 51 patients needed to be included for the multiple regression analyses. A multiple repeated measure regression (repeated over one to four treatment points per patient) was used to establish a predictive model on oral health-related qualitative life with oral lichen planus.

The repeated measure regression was performed with Stata statistical software package version 15 (Stata Corp LCC). Statistical values were considered significant when *P* < 0.05 and highly significant when *P* < 0.001.

## Results

### Collective

The study comprised a total of 53 patients, 46 women (86.8%), and 7 men (13.2%), distributed in following age categories: 21–35 years of age 6 patients, 36–50 years of age 12 patients, 51–65 years of age 19 patients, 66–80 years of age 16 patients. The mean age was 56.5 ± 13.7 years (min. 21–max. 79). The characteristics of the collective are presented in Table 1.

No removable denture was used by 79.2% (*n* = 42), 18.9% (*n* = 10) were wearing a partial denture, and 1.9% had a complete denture (*n* = 1). The mean number of missing teeth was 5.3 ± 5.5 teeth, 18.9% (*n* = 10) had no missing teeth, and 3.8% had 20 missing teeth (*n* = 2). The mean time between the first visit and the last visit was 384 ± 351 days (min. 14–max. 1713 days). The mean time between the first and second visit was 241 ± 220 days (min. 14–max. 945 days). Eighteen patients came for a third visit after a mean period of 269 ± 219 days between the third and second visit (min. 14–max 959). Whereas 7 patients came for a fourth visit after a mean period of 392 ± 413 days between third and fourth visit (min. 91–max. 1234). Eleven patients came before the recommended recall period and 32 after (90–200 days).

The difference between the last and first appointment was as follows:

The mean VAS improved by − 15 ± 29 mm, men showing significantly more improvement (t-2.5, *P* = 0.02).

The mean clinical severity index (CSI) improved by − 1.2 ± 1.2 under therapy. CSI improved significantly by the following factors: age, the number of missing teeth and the personality trait “extraversion.”

In summary for all four appointments, CSI 0 was counted 6 times: CSI 1, 37 counts; CSI 2, 33 counts; CSI 3, 21 counts; CSI 4, 19 counts; CSI 5, 1 count. At the last visit, CSI 4 and 5 were not present while found in 15 patients at the baseline visit (Table 3)

**Table 3:** Different OHIP summary scores (OHIP-G for the German version with 53 questions, OHIP 49 scores, and OHIP 14), subscale scores, and subdimension scores

	1st	2nd	3rd	4th	Mean
OHIP-G	52.7	42.9	26.2	42	45.4
OHIP 49	49.7	40.0	24.7	39.8	42.7
OHIP 14	13.8	11.8	7.1	13.2	12.2
Functional limitation	8.6	8.7	5.4	7.7	8.3
Social disability	3.7	3.9	2.6	3.7	3.7
Handicap	4.3	4.1	2.0	7.2	4.2
Psychological discomfort	8.9	6.3	4.5	5.0	7.33
Psychological disability	6.6	5.2	2.7	9.7	5.7
Physical disability	7.8	7.0	4.4	9.2	7.2
Physical pain	14.1	11.3	9.2	9.8	12.3
Oral function	3.1	3.1	2.0	4.5	3.11
Psychosocial impact	7.0	6.0	4.4	10.3	6.5
Orofacial pain	11.9	9.1	7.7	7.2	10.1
Appearance	5.3	4.2	3.7	1.7	4.7
DT	3.5	3.8	2.2	3.2	3.5

Oral health-related quality of life, assessed by the mean OHIP summary score, improved with the elapsed time in between the visits (t-2.1, *P* = 0.04) (Tables 2 and 3), when hyaluronic acid was used for treatment and the personality trait “conscientiousness” was pronounced. There was a worsening with topical triamcinolone use, with higher CSI scores (Tables 4 and 5).

**Table 4:** Distribution of clinical severity counts at different visits

Visit	CSI	Number of patients
1	1	14
	2	11
	3	9
	4	14
	5	1
2	0	4
	1	15
	2	14
	3	8
	4	4
3	0	1
	1	5
	2	7
	3	2
	4	1
4	0	1
	1	3
	2	1
	3	2

**Table 5:** Multiple regression analysis of significant independent variables on perceived VAS, CSI, and OHIP; *CSI*, clinical severity score; *HA*, hyaluronic acid; *TC*, topical triamcinolone; *SC*, systemic corticosteroids

VAS				
Predictor	Δ*R*^2^	*β*	*P*	*T*
Gender	0.12	− .34	0.016	− 2.509
CSI				
Predictor	Δ*R*^2^	*β*	*P*	*T*
Age	0.14	− .30	0.022	− 2.05
Missing teeth	0.14	− .50	0.007	− 3.55
“Extraversion”	0.14	− .41	0.006	− 2.93
OHIP-G				
Predictor	Δ*R*^2^	*β*	*P*	*T*
CSI	0.40	.40	0.000	4.6
HA	0.40	− .29	0.007	− 2.7
TC	0.40	.32	0.004	3
“Conscientiousness”	0.40	− .22	0.03	− 2.1
Physical pain				
Predictor	Δ*R*^2^	*β*	*P*	*T*
CSI	0.35	.43	0.000	4.8
HA	0.35	− .30	0.008	− 2.7
TC	0.35	.26	0.02	2.3
Physical disability				
Predictor	Δ*R*^2^	*β*	*P*	*T*
CSI	0.42	.42	0.000	4.9
HA	0.42	− .23	0.03	− 2.2
TC	0.42	.32	0.003	3.1
SC	0.42	.20	0.02	2.3
“Conscientiousness”	0.42	− .30	0.003	− 3
Functional limitation				
Predictor	Δ*R*^2^	*β*	*P*	*T*
CSI	0.33	.35	0.000	3.8
TC	0.33	.22	0.05	2
SC	0.33	.21	0.03	2.2
Psychological disability				
Predictor	Δ*R*^2^	*β*	*P*	*T*
CSI	0.35	.30	0.001	3.4
HA	0.35	− .23	0.04	− 2.1
TC	0.35	.26	0.02	2.4
Social disability				
Predictor	Δ*R*^2^	*β*	*P*	*T*
CSI	0.28	.20	0.04	2.1
“Conscientiousness”	0.28	− .29	0.01	− 2.6
Handicap				
Predictor	Δ*R*^2^	*β*	*P*	*T*
CSI	0.36	.21	0.02	2.4
SC	0.36	.23	0.01	2.5
“Conscientiousness”			0.004	− 3
Psychological discomfort				
Predictor	Δ*R*^2^	*β*	*P*	*T*
CSI	0.35	.34	0.000	3.8
HA	0.35	− .31	0.006	− 2.8
TC	0.35	.27	0.02	2.5
Orofacial pain				
Predictor	Δ*R*^2^	*β*	*P*	*T*
CSI	0.35	.44	0.000	4.9
Oral function				
Predictor	Δ*R*^2^	*β*	*P*	*T*
CSI	0.40	.40	0.000	4.6
Missing teeth	0.40	.32	0.01	2.7
SC	0.40	.23	0.01	2.5
PDT	0.40	.19	0.04	2.1
Psychosocial impact				
Predictor	Δ*R*^2^	*β*	*P*	*T*
CSI	0.35	.24	0.01	2.6
HA	0.35	− .28	0.01	− 2.5
TC	0.35	.27	0.02	2.5
“Conscientiousness”	0.35	− .25	0.02	− 2.4
Appearance				
Predictor	Δ*R*^2^	*β*	*P*	*T*
CSI	0.29	.31	0.001	3.3
HA	0.29	− .26	0.03	− 2.3
DT				
Predictor	Δ*R*^2^	*β*	*p*	*T*
TC	0.22	.25	0.04	2

Considering the different treatment options, 86.8% (*n* = 46) of the patients were using hyaluronic acid (Gengigel®) for a mean of 313 ± 350 days three times a day, 52.8% (*n* = 28) topical triamcinolone for a mean of 162 ± 321 days three times a day, 7.5% (*n* = 7) topical tacrolimus for a mean of 21 ± 88 days twice a day, 5.7% (*n* = 3) systemic glucocorticoids for a mean of 18 ± 127 days, 22.6% (*n* = 12) Solcoseryl dental adhaesive® several times a day for a mean of 69 ± 198 days, 3.8% (*n* = 2) cyclosporine mouth rinse (200 mg/twice daily for five minutes) for 19 ± 115 days, and 3.8% photodynamic therapy with 5-aminolevulinic acid once a day for 2 ± 11 days.

The NEO-FFI domains “neuroticism” showed a mean of 19.62 ± 5.97, “extraversion” 27.32 ± 6.21, “openness” 28.65 ± 4.9, “agreeableness” 33.75 ± 4.96, and “conscientiousness” 34.58 ± 5.69.

### OHIP

The clinical severity index (CSI) influenced the OHIP-G summary score, especially the dimensions “physical pain,” “physical disability,” “functional limitation,” “psychological disability,” “social disability,” “handicap,” “psychological discomfort,” “orofacial pain,” “oral function,” “psychosocial impact,” and “appearance.” The worse the clinical presentation is, the higher the impact on perceived daily quality of life (Table 5).

### Considering the influence of different treatment options on OHIP (Table 5)

Hyaluronic acid (Gengigel®) had a significant influence on OHIP improvement, subscore “physical pain,” “psychological discomfort,” “physical disability,” “psychological disability, dimension,” “psychosocial impact, dimension,” “orofacial pain,” and “appearance.”

Topical corticosteroids had a significant negative impact on OHIP-G, “functional limitation,” “psychological disability,” “physical disability,” “psychological discomfort,” “physical pain,” “DT,” and “psychosocial impact.”

Systemic corticosteroids had a negative impact on OHIP-G, subscores “handicap,” “physical disability,” “oral function,” and “functional limitation.”

The photodynamic therapy had a significant influence on the perceived worsening of “oral function” (t-2.1, *P* = 0.04).

### NEO-FFI

“Neuroticism” 1.6 ± 0.5, “extraversion” 2.3 ± 0.5, “openness” 2.4 ± 0.4, “agreeableness” 2.8 ± 0.4, and “conscientiousness” 2.9 ± 0.5.

The facet “conscientiousness” had a significant influence on OHIP-G, subscore improvement “social disability,” “handicap,” “physical disability,” “DT,” and “psychosocial impact” (Table 5).

A pronounced “extraversion” personality trait significantly influenced the clinical presentation assessed by CSI (*P* = 0.006) during the course of treatment.

## Discussion

A recent Cochrane review [21] quoted that the impact of pain on physical, emotional, and social functions required multidimensional qualitative tools and health-related quality of life instruments that are uncommonly used in OLP trials. Due to the chronic nature of OLP and the impact of this condition on quality of life, the use of patient-reported outcome measures in assessing the treatment of chronic oral mucosal diseases is of great importance. The influence of personality traits on oral health-related quality of life in patients suffering from the mucosal disease has been described previously [8]. Moreover, the present follow-up study showed a significant impact of personality traits on therapeutic outcomes in oral lichen planus patients. The facets “extraversion” and “conscientiousness” showed a significant influence on subjective as well as objective disease outcomes.

This chronic mucosal disease is mainly prevalent in a middle-aged female population where personality traits have been reported to be more stable after 30 years of age. The neuroticism extraversion openness five-factor inventory (NEO-FFI-R) questionnaire evaluates five personality traits (i.e., neuroticism, extraversion, openness, agreeableness, and conscientiousness) and consists of a psychological test with good structure and reliability. The trait “conscientiousness” had a significant influence on oral health-related quality of life in physical and psychosocial aspects. This trait is characterized by a tendency to be organized, show self-discipline, act dutifully, aim for achievement, and prefer planned rather than spontaneous behavior. Also, in recent literature, conscientiousness was positively related to treatment adherence, as this trait estimates motivation in goal-directed behavior. Regularity is required in topical treatment with immunomodulating agents. Therefore, the facet “conscientiousness” could influence higher treatment adherence. Furthermore, the caregiver’s conscientiousness could have a significant influence on treatment outcome [22]. Self-efficacy mediated the effect of extraversion and conscientiousness on health-related quality of life [23–25].

The trait “extraversion” had a significant influence on the clinical severity of OLP patients. Extraversion is characterized by gratification from the external environment of the patient, linking interaction with others, being full of energy in the presence of others, and prefers human companionship to being on their own. Whether chronic disease may influence personality traits is a matter of debate. Nevertheless, clinically more severe cases may reduce the tendency to seek stimulation in the company of others and talkativeness. “Extraversion” seems to be protective in the psychosocial domain. This trait includes characteristics such as excitability, sociability, assertiveness, and high amounts of emotional expressiveness. Thus, an improvement in clinical severity may influence the sociability and talkativeness of patients. Vice versa, “extraversion” may modulate cortisol response to stress in chronic disease. It is known that OLP can appear and worsen during stressful events. Psychosocial factors such as personality traits are significantly associated with quality of life ratings. Such associations should be considered when the quality of life measurements are used and interpreted. Certain personality traits can lead to predicting positive health outcomes. In an elderly Japanese sample, conscientiousness, extraversion, and openness were related to a lower risk of mortality [26]. Individuals that score high on extraversion are prone to a more physically active lifestyle. In addition, extraversion negatively predicts perceived depression levels and potentially perceived stress. Hence, stress has a significant influence on OLP scores [27, 28], yet subjective outcome parameters were also influenced by an individual’s characteristics such as male gender and higher age. Furthermore, different therapeutic options have diverse influences on the perceived quality of life. While corticosteroids seem to have a negative impact on well-being, hyaluronic acid has a significant beneficial effect on oral health-related quality of life.

A recent Cochrane review [21] found no evidence of a difference between topical steroids and placebo when measuring clinical resolution. The overall risk ratio of experiencing adverse effects caused by topical steroids was 1.48 compared with the placebo. Low-certainty evidence suggested that corticosteroids, particularly in topical formulations with adhesive bases, were effective in controlling the pain of OLP, though the findings were inconclusive for improving clinical presentation and uncertain about adverse effects.

We must also consider that many patients have prejudices again corticosteroids and patients as well as doctors have not been blinded to the prescribed treatments in the present study. This could have affected the regularity of the application. Furthermore, a strong weakness of the present study is the lack of a control group receiving a placebo or no treatment.

Topical steroids remain the first-line treatment for symptomatic OLP with proven efficacy and safety, but topical agents can probably not remain on the oral mucosa sufficient time to be absorbed. Therefore, the systemic or intralesional application is indicated when topical corticosteroid treatment is insufficient. Corticosteroid treatments nevertheless raise concerns in many patients due to the possible side effects [29]. One side effect of transient burning sensation with relapse has been reported in long-term corticosteroid and calcineurin inhibitor use, as seen especially for the seven patients at the fourth visit.

A recent study showed that 0.2% hyaluronic acid effectively improved the symptoms associated with OLP even after cessation of treatment. Hyaluronic acid is an effective substitute for topical corticosteroid with similar results. Moreover, in severely painful OLP, 0.2% HA could be used in addition to other topical drugs, such as corticosteroids and calcineurin inhibitors, to reduce the overall amount of immunosuppressant therapy used in a course of treatment [30].

Additionally, topical treatments depend on the patient’s compliance, which may influence results. As mentioned previously “conscientiousness” correlates with self-discipline and could influence compliance. The personality factors “conscientiousness” and “agreeableness” were presented as predictors of high treatment adherence in oncology, recently [31]. Results from a renal dialysis study indicated that “conscientiousness” is a five-factor trait significantly associated with adherence to the medication regimen [32]. Personality traits may be a predictor of treatment non-adherence. In an antibiotic use study, the facet “neuroticism” was identified as a negative predictor, and both “agreeableness” and “conscientiousness” were identified as positive predictors of adherence behavior [33]. Hence, as topical therapy options necessitate patients compliance, compliance independent therapy approaches such as intralesional steroid application might be a better option in patients with a low “conscientiousness.”

Concluding, “conscientiousness” and “extraversion” have a significant influence on therapeutic outcomes in OLP patients mainly treated with different topical agents.

Assessment of personality factors may be helpful in predicting the patient’s cooperation and may affect treatment. Therefore, it would be reasonable to assess personality traits, in order to be more aware of patients’ expectations and their possible compliance with an offered treatment. This may avoid extra time and effort. Psychological facets play a role in OLP management and explain the impacts on daily living and patients’ satisfaction.

## References

[1] Zuo W, Li X, Chen Y, Peng H (2012). Oral health-related quality of life in patients with oral lichen planus. Hua Xi Kou Qiang Yi Xue Za Zhi.

[2] Lopez-Jornet P, Camacho-Alonso F (2010). Quality of life in patients with oral lichen planus. J Eval Clin Pract.

[3] Lodi G, Carrozzo M, Furness S, Thongprasom K (2012) Interventions for treating oral lichen planus. Br J Dermatol. 10.1111/j.1365-2133.2012.10821.x 10.1111/j.1365-2133.2012.10821.x 22242640

[4] Lopez-Jornet P, Camacho-Alonso F, Salazar-Sanchez N (2010). Topical tacrolimus and pimecrolimus in the treatment of oral lichen planus: an update. J Oral Pathol Med.

[5] Thongprasom K, Carrozzo M, Furness S, Lodi G (2011) Interventions for treating oral lichen planus. Cochrane Database Syst Rev:CD001168. 10.1002/14651858.CD001168.pub2 10.1002/14651858.CD001168.pub2 21735381

[6] van der Waal I (2009). Potentially malignant disorders of the oral and oropharyngeal mucosa; terminology, classification and present concepts of management. Oral Oncol.

[7] Riordain RN, McCreary C (2012). Validity and reliability of a newly developed quality of life questionnaire for patients with chronic oral mucosal diseases. J Oral Pathol Med.

[8] Fadler A, Hartmann T, Bernhart T, Monshi B, Rappersberger K, Hof M, Dvorak G (2015). Effect of personality traits on the oral health-related quality of life in patients with oral mucosal disease. Clin Oral Investig.

[9] Al-Omiri MK, Karasneh J, Alhijawi MM, Zwiri AM, Scully C, Lynch E (2015). Recurrent aphthous stomatitis (RAS): a preliminary within-subject study of quality of life, oral health impacts and personality profiles. J Oral Pathol Med.

[10] Karasneh J, Al-Omiri MK, Al-Hamad KQ, Al Quran FA (2009). Relationship between patients’ oral health-related quality of life, satisfaction with dentition, and personality profiles. J Contemp Dent Pract.

[11] Torres BL, Costa FO, Modena CM, Cota LO, Cortes MI, Seraidarian PI (2011). Association between personality traits and quality of life in patients treated with conventional mandibular dentures or implant-supported overdentures. J Oral Rehabil.

[12] Penacoba C, Gonzalez MJ, Santos N, Romero M (2014). Psychosocial predictors of affect in adult patients undergoing orthodontic treatment. Eur J Orthod.

[13] Menassa M, de Grandmont P, Audy N, Durand R, Rompre P, Emami E (2016). Patients’ expectations, satisfaction, and quality of life with immediate loading protocol. Clin Oral Implants Res.

[14] John MT, Patrick DL, Slade GD (2002). The German version of the Oral Health Impact Profile--translation and psychometric properties. Eur J Oral Sci.

[15] John MT, Micheelis W, Biffar R (2004). Reference values in oral health-related quality of life for the abbreviated version of the Oral Health Impact Profile. Schweiz Monatsschr Zahnmed.

[16] Salazar-Sanchez N, Lopez-Jornet P, Camacho-Alonso F, Sanchez-Siles M (2010). Efficacy of topical Aloe vera in patients with oral lichen planus: a randomized double-blind study. J Oral Pathol Med.

[17] Abu Hantash RO, Al-Omiri MK, Al-Wahadni AM (2006). Psychological impact on implant patients’ oral health-related quality of life. Clin Oral Implants Res.

[18] Thongprasom K, Chaimusig M, Korkij W, Sererat T, Luangjarmekorn L, Rojwattanasirivej S (2007). A randomized-controlled trial to compare topical cyclosporin with triamcinolone acetonide for the treatment of oral lichen planus. J Oral Pathol Med.

[19] Amin KA, Abdel Hameid H, Abd Elsttar AH (2010). Effect of food azo dyes tartrazine and carmoisine on biochemical parameters related to renal, hepatic function and oxidative stress biomarkers in young male rats. Food Chemi Toxicol.

[20] Thong ISK, Jensen MP, Miro J, Tan G (2018). The validity of pain intensity measures: what do the NRS, VAS, VRS, and FPS-R measure?. Scand J Pain.

[21] Lodi G, Manfredi M, Mercadante V, Murphy R, Carrozzo M (2020). Interventions for treating oral lichen planus: corticosteroid therapies. Cochrane Database Syst Rev.

[22] Ma X, Meng G, Tan Y, Liu X, Zhao Y, Yu J, Jin A, Zhao Y, Liu X (2018). Patient and family caregiver’s neuroticism and conscientiousness personality in relation to quality of life of patient with Parkinson’s disease: a cross-sectional study neuroticism and conscientiousness personality in relation to QoL of patient with PD. Front Neurol.

[23] Axelsson M, Brink E, Lundgren J, Lotvall J (2011). The influence of personality traits on reported adherence to medication in individuals with chronic disease: an epidemiological study in West Sweden. PLoS One.

[24] Axelsson M, Lotvall J, Cliffordson C, Lundgren J, Brink E (2013). Self-efficacy and adherence as mediating factors between personality traits and health-related quality of life. Qual Life Res Int J Qual Life Asp Treat Care Rehab.

[25] Axelsson M, Cliffordson C, Lundback B, Lotvall J (2013). The function of medication beliefs as mediators between personality traits and adherence behavior in people with asthma. Patient Prefer Adherence.

[26] Iwasa H, Masui Y, Gondo Y, Inagaki H, Kawaai C, Suzuki T (2008). Personality and all-cause mortality among older adults dwelling in a Japanese community: a five-year population-based prospective cohort study. Am J Geriatr Psychiatry.

[27] Mohamadi Hasel K, Besharat MA, Abdolhoseini A, Alaei Nasab S, Niknam S (2013). Relationships of personality factors to perceived stress, depression, and oral lichen planus severity. Int J Behav Med.

[28] Cerqueira JDM, Moura JR, Arsati F, Lima-Arsati YBO, Bittencourt RA, Freitas VS (2018). Psychological disorders and oral lichen planus: a systematic review. J Investig Clin Dent.

[29] Kurt MH, Kolsuz ME, Eren H (2019). Corticosteroid injection in treatment of persistent oral lichen planus: three cases. Dermatol Ther.

[30] Hashem AS, Issrani R, Elsayed TEE, Prabhu N (2019). Topical hyaluronic acid in the management of oral lichen planus: a comparative study. J Investig Clin Dent.

[31] Lima MP, Machado WL, Irigaray TQ (2018). Predictive factors of treatment adherence in cancer outpatients. Psycho-oncology.

[32] Christensen AJ, Smith TW (1995). Personality and patient adherence: correlates of the five-factor model in renal dialysis. J Behav Med.

[33] Axelsson M (2013). Report on personality and adherence to antibiotic therapy: a population-based study. BMC Psychol.

